# Evidence for Two Types of Task Conflict in a Color-Digit Stroop Task

**DOI:** 10.5334/joc.386

**Published:** 2024-07-12

**Authors:** Ronen Hershman, Eldad Keha, Ayelet Sapir, Elisabeth M. Weiss, Avishai Henik, Liane Kaufmann

**Affiliations:** 1 Department of Psychology, University of Innsbruck, Innsbruck, Austria; 2 Department of Psychology, The Hebrew University, Jerusalem, Israel; 3 Department of Psychology, Achva Academic College, Beer-Tuvia, Israel; 4 School of Psychology and Sport Science, Bangor University, Bangor, United Kingdom; 5 Department of Psychology and The Zelman Center for Brain Science, Ben-Gurion University of the Negev, Beer-Sheva, Israel; 6 Department of Neurology and Clinical Neuropsychology, Ernst von Bergmann Klinikum Potsdam, Potsdam, Germany

**Keywords:** numerical cognition, Stroop effect, task conflict, stimulus-stimulus conflict, stimulus-response conflict

## Abstract

In the present study, we conducted a Stroop-like task in which the participants were required to decide whether the presented stimulus, which could be either a colored digit or a colored rectangle, consisted of more or less than five colors. Like other Stroop-like tasks, the stimuli could be congruent (the stimulus was a digit that was equal to the presented number of colors), incongruent (the stimulus was a digit that was different than the presented number of colors), or neutral (a colored rectangle). We utilized a two-to-one response setting so that in some incongruent trials the digit and the number of colors would elicit the same response (e.g., the digit 3 containing two colors; both are smaller than 5), while in some incongruent trials, the digit and the number of colors would elicit different responses (e.g., the digit 3 containing 6 colors). This enabled us to measure both conflicts arising from stimulus-stimulus and stimulus-response compatibilities. Our results indicated the existence of stimulus-stimulus compatibility (SSC), stimulus-response compatibility (SRC), and task conflict. Interestingly, these effects were in interaction with the number of colors, so that in small numbers, SSC and SRC were found, and in large numbers, SRC and task conflict were found. Moreover, the results suggest that our task includes two types of task conflict that are raised due to three different tasks: processing the meaning of the digit vs. estimating the number of colors and counting the number of colors vs. estimating the number of colors.

Cognitive control often refers to our ability to focus on relevant and ignore irrelevant information. One of the commonly used tasks to examine these abilities is the color-word Stroop task (Stroop, 1935), where participants are presented with colored stimuli and are asked to name their color as fast and accurately as possible. In most cases, the presented stimuli are color words or stimuli associated with color words (e.g., Henik et al., 2018; Parris et al., 2023). It is often found that the incongruent condition, in which the meaning of the word is different from the color of the stimuli (e.g., the word RED printed in blue), yields a slower reaction time (RT) as compared to neutral non-color word stimuli (as well as any cluster of colored pixels). This slowdown of RTs is also referred to as the interference effect. In addition, if the written word is congruent with the ink color of the stimulus (e.g., RED written in red), the RT for these stimuli is usually faster than for neutral stimuli. This acceleration in RT is called the facilitation effect, and compared to the interference effect, it is usually smaller and more fragile (Henik et al., 2018; Hershman & Henik, 2019; Kalanthroff & Henik, 2013).

Recently, there has been a growing interest in the different levels of processing that take place in the Stroop task (Augustinova et al., 2019; Parris et al., 2023). Two main conflicts are usually reported: (i) the information conflict, which represents a conflict between two pieces of information (i.e., the color of the word and the meaning of the word), and (ii) the task conflict, which represents a conflict with the irrelevant task of reading.

In general, information conflict is typically explored by comparing incongruent trials (with conflicting information) to congruent trials (with matching information). Task conflict is often measured by comparing congruent trials (where two possible tasks are triggered) to non-word neutral trials (where only one relevant task is triggered). Under certain conditions, this may result in slower RTs for congruent compared to non-word neutral trials – the reverse facilitation effect (Kalanthroff et al., 2018; Keha & Kalanthroff, 2023).

The idea of task conflict rests on the premise that stimuli evoke prepotent task tendencies that are strongly associated with them (Rogers & Monsell, 1995; Waszak et al., 2003). Words tend to evoke reading (Monsell et al., 2001), and, as a result, when one has to name the color of the ink, word reading competes with color naming and creates task conflict. Interestingly, it has been suggested that the information conflict can result from two distinct processes: differences in stimulus-response conflict (SRC), which elicits conflict between the response to the color and the response to the word; and a stimulus–stimulus conflict (SSC), which elicits conflict between two contradictive pieces of information: the color and the meaning of the stimulus (Chen et al., 2011; De Houwer, 2003; Graf et al., 1984; Hasshim & Parris, 2015; Hershman & Henik, 2020; Shichel & Tzelgov, 2018; van Veen & Carter, 2005).

De Houwer’s (2003) two-to-one Stroop task is one effective way to disentangle informational conflict into its components. In this task, multiple ink colors are mapped to one response key. For example, four colors—red, blue, green, and yellow—are presented to participants, but there are only two response keys such that the colors red and blue are assigned to the “M” key, while the colors yellow and green are assigned to the “C” key. A two-to-one task produces four possible conditions: neutral (represented by non-color words), congruent (represented by color words whose meaning and ink color are the same), and two incongruent conditions. One, where the word meaning and ink color are different, but the response is the same (e.g., “BLUE” in red in the given example, where both require the response “M”), and the second, where the word meaning and the ink color are different, and the response is also different (e.g., “BLUE” in green in the given example). According to the terminology suggested above, there are two possible sources of conflict: the SRC indicated by the incongruent trials with a different response (BLUE in green) and the SSC indicated by the incongruent trials with the same response (BLUE in red). That is, the comparison between incongruent trials with a different response and incongruent trials with the same response might reveal the existence of SRC, while the comparison between incongruent trials with the same response and congruent trials might reveal the existence of SSC.

Conflicts from the irrelevant dimension are not limited to written words but can also be found with other Stroop-like tasks. Hershman, Keha, et al. (2024) developed a novel numeric Stroop-like task and showed that the mere presence of numerical information within the irrelevant dimension is sufficient to trigger a conflict from the spontaneous activation of the digits’ numerical value. In this task, participants were presented with stimuli that consisted of horizontal stripes of colors and were asked to report how many colors the stimuli consisted of. The stimuli were presented sequentially (i.e., one at a time) and could either be colored rectangles (i.e., the neutral condition) or colored digits. Similar to the other Stroop-like tasks, there are both congruent trials, where the number of colors is identical to the numerical value of the stimulus (e.g., the digit 3 consisting of three colors), and incongruent trials, where the number of colors is different from the numerical value of the stimulus (e.g., the digit 3 consisting of two colors). Accordingly, both interference and facilitation were found in this task. Moreover, pupillometry data also confirmed that in this experiment, both task and information conflicts could be distinguished (Hershman, Beckmann, et al., 2024). Note that in the color-digit Stroop task (Hershman, Keha, et al., 2024), the irrelevant dimension is the numerical value of the digit, and the relevant dimension is the quantity of the presented colors.

In the original color-digit Stroop task (Hershman, Keha, et al., 2024), participants were asked to respond by pressing one of four keys to determine the number of colors within the stimuli. This response setting does not allow a dissociation between the SSC and SRC. To address these types of conflicts, one should use a two-to-one response mapping (De Houwer, 2003), where, as described above, some incongruent stimuli are associated with the same response as the required response, and some are associated with a different response. Therefore, in the current study, we used two-to-one response setting to allow the measure of SSC and SRC separately. We did it using small and large numbers (and quantities) in the color-digit Stroop task.

Processing of quantities brings along further effects; for quantities smaller than around 4 or 5, observers employ a precise judgment. This can be either done by counting or via the process of subitizing (a quick and quasi-automatic precise measurement of small quantities – Starkey & Cooper, 1980; Trick & Pylyshyn, 1994). In contrast, for larger quantities, observers may employ estimation processes, probably using the Approximate Number System (Dehaene, 1997). Interestingly, using functional magnetic resonance imaging (fMRI), it was found that exact and approximate calculations resulted in different brain activation patterns in distinct regions (Stanescu-Cosson et al., 2000). Specifically, exact calculations involved a left-lateralized inferior frontal region, while approximate calculations yielded bilateral activation in the frontal and parietal networks.

Given the difference in small and large quantities and the distinct processes associated with judgments of precise numbers and those associated with the estimation of approximate numbers, it is not unreasonable to expect that different processes may manifest when using small and large numbers in the color-digit Stroop task. For example, when participants employ a precise evaluation of the number of colors, conflicts related to SRC and also those related to SSC may occur. However, when participants employ an approximate estimation of quantity, only SRC will be present, as the number of colors is estimated as “large” rather than the exact quantity.

In the same way, in the case of a rough estimation of the number (compared to exact quantity), participants may not engage with the irrelevant dimension (the numerical meaning of the stimulus) as much as when employing a precise judgment of quantity. Hence, the employment of precise vs. approximate judgment may affect task conflict as well. If rough estimation causes less processing of the exact number of colors than precise evaluation, the processing of the neutral trials should be faster when the number of colors is larger than 5, and therefore, reverse facilitation (an indicator for task conflict) is expected in large but not small numbers.

## The Current Study

In the present study, we aimed to dissociate task conflict, as well as SSC and SRC, using small and large numbers in a color-digit Stroop-like task (Hershman, Keha, et al., 2024). Participants were presented with objects that consisted of colored horizontal slices and were asked to determine whether the number of colors was smaller or larger than 5. The stimuli could be neutral (colored rectangles), congruent (digits with the same number of colors), or incongruent (digits with a different number of colors). Note that in such a task, the incongruent stimuli could require a similar response to the irrelevant numerical value of the digit (incongruent trials with the same response) or require a different response to the irrelevant numerical value of the digit (incongruent trials with a different response; see an example of the stimuli in Table 1).

**Table 1:** Examples for the Different Stimuli Conditions in the Current Experiment.


CONDITION	SMALLER THAN 5	LARGER THAN 5

Congruent	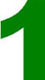	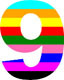

Neutral	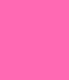	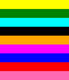

Incongruent with the same response	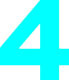	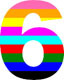

Incongruent with a different response	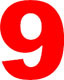	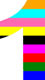


We predicted that SSC and SRC would be different for the smaller (than 5) and the larger (than 5) number of colors. Specifically, we expected both SSC and SRC to appear in the smaller than 5 condition, but in the larger than 5 condition, only SRC was expected. In the smaller than 5 colors, the correct number of colors (i.e., one to four) can be perceived quasi simultaneously (i.e., the subitizing account: Starkey & Cooper, 1980; Trick & Pylyshyn, 1994). Hence, when the irrelevant digit is, for instance, 3, and the number of colors is two, the conflict between these two pieces of information would give rise to SSC. While the response is unmistakably “smaller than 5”, there is still a noticeable conflict due to the precise determination of the number of colors. In contrast, in the larger than 5 colors, the correct number of colors (i.e., six to nine) would only be roughly estimated as “large” or “many”, because precise counting might be effortful (for a review, see Mix et al., 2002; Trick & Pylyshyn, 1994). Accordingly, when the irrelevant digit is, for instance, 7, and the number of colors is six, they are both considered “large”, and this difference might not create an SSC. If this expectation is reflected in the results, it would suggest that for SSC to appear, the pieces of information provided by the relevant and irrelevant dimensions should be very clearly perceived.

One interesting question in the current study is related to the neutral trials. If, indeed, participants use precise judgment in small quantities and crude estimation in large quantities, one can expect this to be shown in neutral trials as well. Therefore, neutral trials containing a large number of colors may be processed faster than those containing a small number of colors simply because participants do not engage in precise judgment. This could result in a smaller facilitation effect in large numbers compared to small numbers or even a reverse facilitation (an indicator of task conflict).

## Method

### Participants

Power analysis for the sample size was calculated using MorePower 6.0 (Campbell & Thompson, 2012) for the within 4 × 2 repeated measures interaction with an effect size of partial eta squared ( \[\eta _p^2\]) = 0.16 based on results from a similar design (Parris, 2014) that tested a similar interaction for different types of conflict. To achieve a power of 99% the required sample size was 44. Since we ran this experiment online, we recruited more participants to ensure that after dropping participants from the pool, we still had a sufficient sample size. Eventually, fifty-four students (33 females and 21 males, mean age 24 years, *SD* = 3) from the University of Innsbruck participated in the experiment in return for fulfillment of course requirements.

The study was approved by the board for ethical questions in science at the University of Innsbruck (77/2021). None of the participants reported color blindness or the presence of a diagnosed neurological and/or psychiatric disorder (including attention disorders or learning disabilities).

### Stimuli

The stimuli were colored single-digit numbers (Arial font), which were based on the stimuli of Hershman, Keha, et al.’s (2024) set of stimuli (all the presented stimuli are free and available here: https://osf.io/g2jau/?view_only=9aeca2f8736b414398d1fb11eda34e60). The digits that were used were 1, 2, 3, 4, 6, 7, 8, and 9, in a size of 640 × 740 pixels. The single-digit could consist of a different number of colors between one to nine (except five) colors (red: RGB = 255, 0, 0; blue: RGB = 0, 0, 255; green: RGB = 0, 130, 0; yellow: RGB = 255, 255, 0; magenta: RGB = 255, 0, 255; cyan: RGB = 0, 255, 255; orange: RGB = 255, 165, 0; pink: RGB = 255, 105, 180; and black: RGB = 0, 0, 0). The stimuli were cut horizontally into parts of equal height according to the required number of colors. In addition to the presented colored single-digit numbers, colored rectangles of the same size and colors (see Table 1) were used as neutral stimuli. The presented stimuli (see Table 1 for an example) appeared against a white (RGB: 255, 255, 255) background.

### Procedure

Participants were tested online by using *Minno. js instead* (Zlotnick et al., 2015) on their own electronic devices. The program required a spacebar response, ensuring participants only used computers rather than tablets or mobile phones. Participants were asked to decide whether the number of colors in the stimulus was smaller or larger than 5. In total, the stimuli created eight possible conditions. First, there were two possible decisions: smaller or larger than 5. Second, for each possible decision, there were four options for congruency. In addition to the congruent trials (i.e., the numerical value of the presented stimulus was the same as the number of colors it consisted of), and the neutral trials (i.e., rectangles without a numerical value, which have already been suggested to be preferred over other types of neutrals because they include no meaning that might be processed (Hershman et al., 2021)), there were two kinds of incongruent trials: incongruent trials with the same response (i.e., the numerical value of the presented stimuli was different from the number of colors it consisted of, but the answer was identical, e.g., the digit 3 composed of two colors) that might indicate SSC, and incongruent trials with a different response (i.e., both the answer and the numerical value of the presented stimuli were different from the number of colors it consisted of, e.g., the digit 3 composed of nine colors) that might indicate SRC. The conditions and the stimuli for each participant were selected randomly but were balanced.

**Figure 1:**
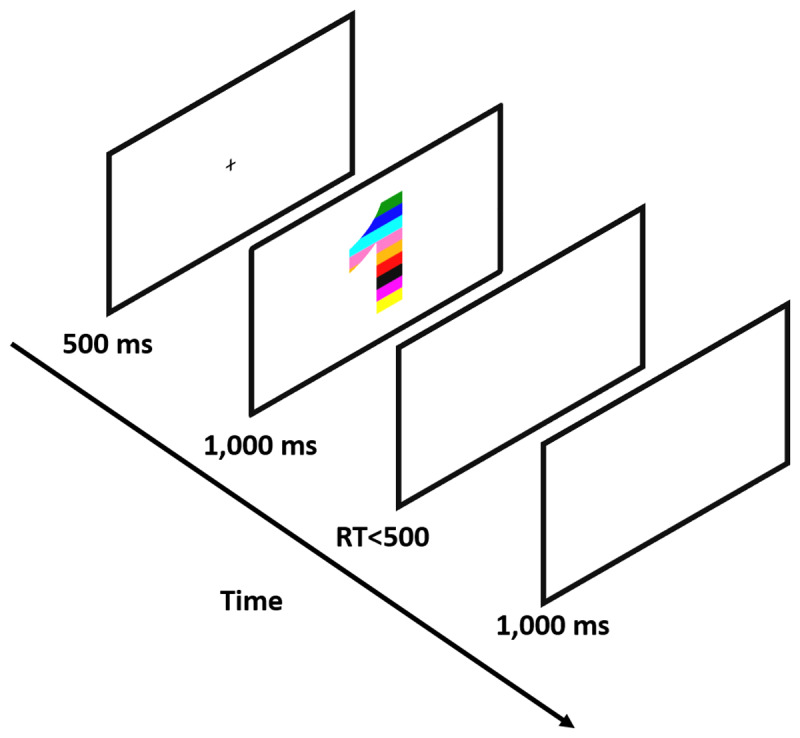
An Example of An Incongruent Trial. *Note*. Participants were required to report whether the number of colors that painted the presented stimulus was smaller or larger than 5.

The experiment included 10 practice trials that were excluded from the analysis. After each practice trial, participants received feedback on their accuracy. Participants were required to achieve at least 80% correct trials in practice to proceed to the experimental part (i.e., at least eight correct responses), or otherwise, the practice session was presented again. In the experimental part, participants carried out 400 experimental trials (50 for each congruency condition for each group of numbers; smaller/larger than 5). At the beginning of each trial (see Figure 1 for a visual depiction), there was a black fixation cross presented for 500 ms at the center of the screen. The fixation was followed by a visual stimulus that appeared on the screen for 1,000 ms and was followed by a blank screen for a maximum of 500 ms or until a keypress. Each trial ended with a 1,000 ms inter-trial interval (ITI) of a blank (white) screen. Participants were asked to hit the “C” key if the stimulus was painted with less than five colors and the “M” key if the stimulus was painted with more than five colors (the key mapping was counterbalanced across participants). RT was calculated from the appearance of the visual stimulus to the reaction in the form of a key press.

### Design

This was a within-subject experiment, where RT was the dependent variable, and the congruency condition and the number of colors were the independent variables. Congruency included four levels (congruent, incongruent trials with the same response, incongruent trials with a different response, and neutral), and the number of colors included two levels (less than 5 and more than 5).

## Results

Seven participants were excluded from the analysis due to their accuracy being less than 75% in each condition.

In our analyses, in addition to classical null hypothesis significance testing (NHST), we used Bayesian statistics using JASP (statistical software; JASP Team, 2017) with the default recommended parameters. The Bayesian statistics provided Bayes factors (BF; Jeffreys, 1961). *BF*_10_ means evidence favors the alternative hypothesis against the null hypothesis. Values above 3 are considered moderate evidence supporting the alternative hypothesis, values above 10 are considered strong support, values above 30 are considered very strong support and values above 100 are considered extreme support. Conversely, *BF*_01_ means evidence favors the null hypothesis against the alternative hypothesis.

For the Bayesian repeated-measure analysis of variance (ANOVA) tests, we used BF inclusion, which was given by JASP. BF inclusion compares the relative support for two models, one including a particular factor of interest and one without. Conceptually, BF inclusion helps to see if the data makes a particular factor more or less important for explaining the results, considering all possible models with and without that factor.

### Accuracy

**Figure 2:**
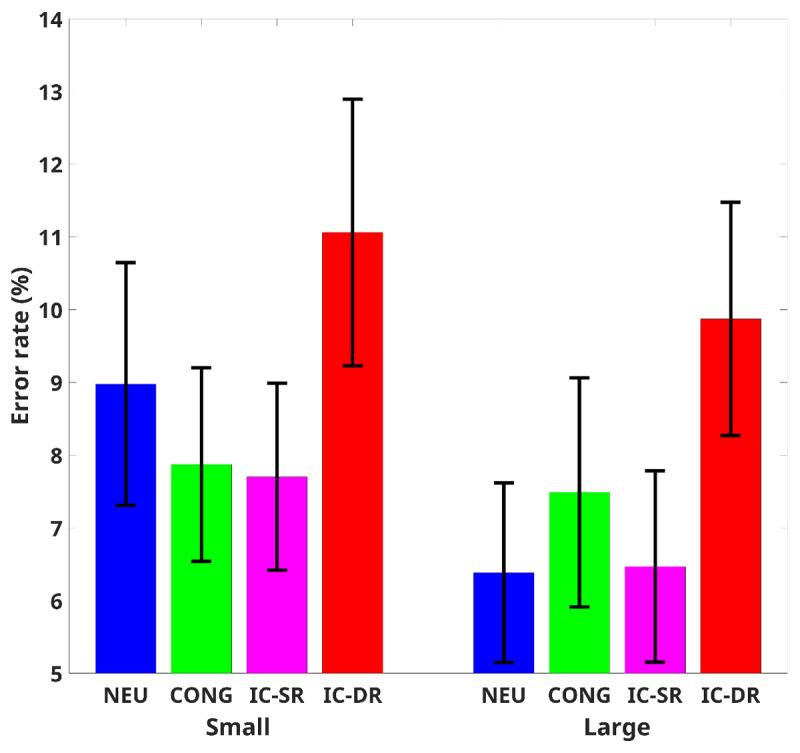
Error Rate for Each Congruency Condition. *Note*. Cong = congruent, Neu = neutral, IC – SR = incongruent – same response, IC – DR = incongruent – different response, Small = numbers smaller than 5, Large = numbers larger than 5. Error bars represent 95% confidence interval from the mean.

Mean accuracy for each participant in each condition was subjected to a two-way repeated-measures ANOVA with congruency (congruent, neutral, incongruent trials with the same response, and incongruent trials with a different response) and the number of colors (smaller than 5, larger than 5) as independent factors (mean RTs in the various conditions are presented in Figure 2). Our analysis showed a meaningful (*BF*_10_ ≥ 3) higher accuracy for a large number of colors than a small number of colors, *F*(1,46) = 7.93, *p* = .007, \[\eta _p^2\] = 0.15, *BF*_inc_ = 3.24, and for congruency, *F*(3,138) = 15.27, *p* < .001, \[\eta _p^2\] = 0.25, *BF*_inc_ > 10^4^. No interaction was found between congruency and the number of colors, *F*(3,138) = 1.22, *p* = .31, \[\eta _p^2\] = 0.03, *BF*_inc_ = 0.17 ≡ *BF*_01_ = 6.06.

Post-hoc analysis comparisons showed that incongruent trials with a different response were less accurate than incongruent trials with the same response (*t*(46) = 5.96, *p*_holm_ < .001, *BF*_10_ > 10^6^, *Cohen’s d* = .86), congruent (*t*(46) = 5.09, *pholm* < .001, *BF*_10_ > 1,000, *Cohen’s d* = .63), and neutral (*t*(46) = 5.27, *p*_holm_ < .001, *BF*_10_ > 1,000, *Cohen’s d* = .14) trials. No meaningful differences were found between all the other conditions (*p* > .05, *BF*_10_ < 0.3 ≡ *BF*_01_ > 3).

### Reaction time

**Figure 3:**
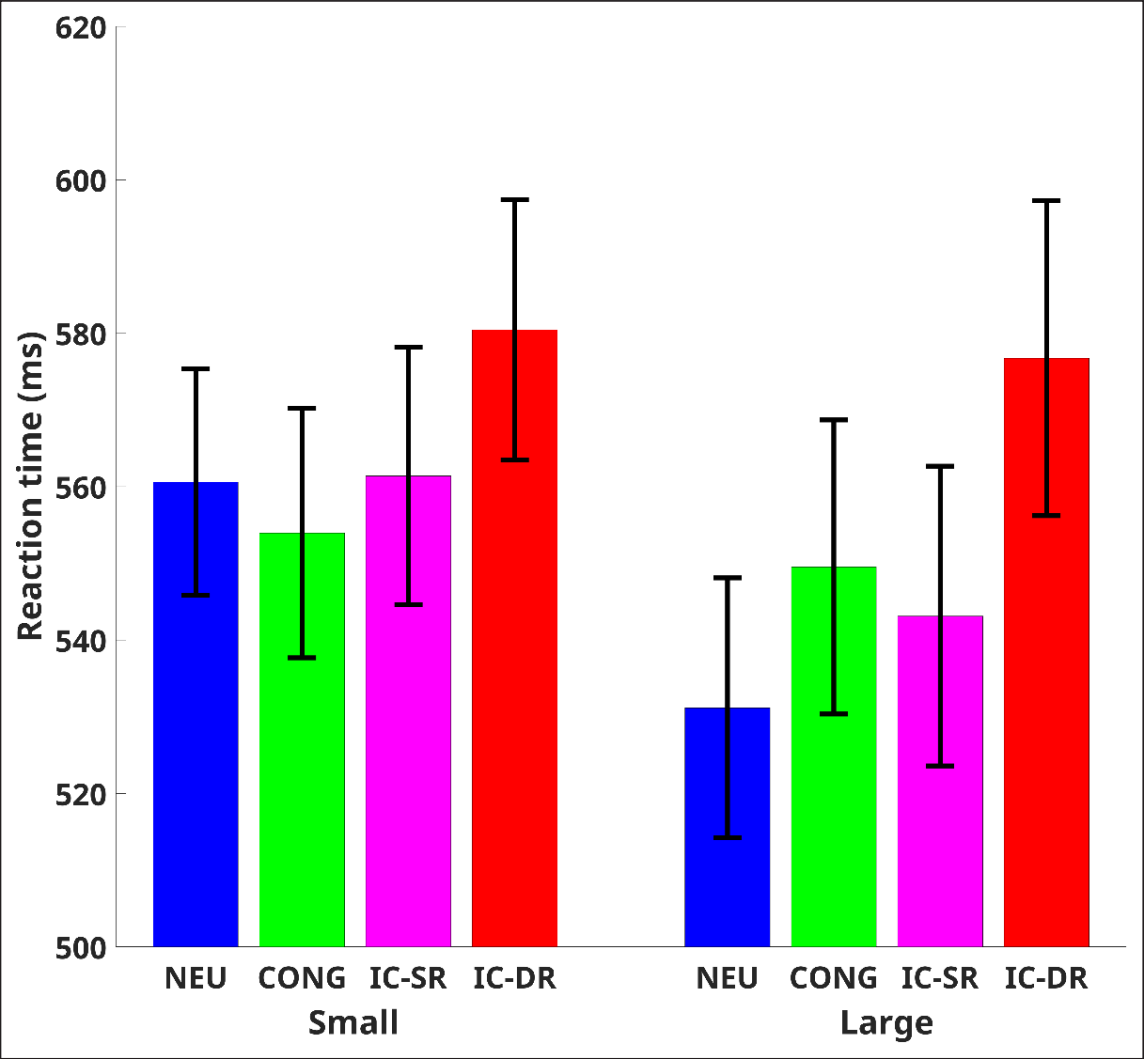
Mean Reaction Time for Each Congruency Condition. *Note*. Cong = congruent, Neu = neutral, IC – SR = incongruent – same response, IC – DR = incongruent – different response, Small = numbers smaller than 5, Large = numbers larger than 5. Error bars represent 95% confidence interval from the mean.

Seven participants were excluded from the analysis due to their accuracy being less than 75% in each condition. For each participant, mean RTs and standard deviations were calculated separately across all the experimental trials. Then, extremely slow and fast responses were excluded from the analysis (RTs larger or smaller than 3.5 z-scores from the mean of each subject for each condition separately). Mean RTs of correct response trials for each participant in each condition were subjected to a two-way repeated-measures ANOVA with congruency (congruent, neutral, incongruent trials with the same response, and incongruent trials with a different response) and the number of colors (smaller than 5, larger than 5) as independent factors (mean RTs in the various conditions are presented in Figure 3). As expected, our analysis produced a meaningful (*BF*_10_ ≥ 3) main effect for the number of colors, *F*(1,46) = 5.5, *p* = .023, \[\eta _p^2\] = 0.11, *BFinc* > 1,000, and for congruency, *F*(3,138) = 59.6, *p* < .001, \[\eta _p^2\] = 0.564, *BF*_inc_ > 10^12^ Most importantly, a meaningful interaction was found between congruency and the number of colors, *F*(3,138) = 12.71, *p* < .001, \[\eta _p^2\] = 0.217, *BF*_inc_ = 9.38.

Post-hoc analysis comparisons of differences in the Stroop effects between the number of colors showed meaningful differences. Specifically, the difference between the facilitation (i.e., faster responses for congruent than neutral trials) in a small number of colors and the reverse facilitation (i.e., slower responses for congruent than neutral trials) in a large number of colors was meaningful, *t*(46) = 5.13, *p* < .001, *pholm* < .001, *BF*_10_ > 1,000, *Cohen’s d* = .75. In addition, the difference between congruent trials and incongruent trials with the same response was larger in the small number of colors as compared to large numbers of colors, *t*(46) = 3.15, *p* = .002, *pholm* = .005, *BF*_10_ = 11.55, *Cohen’s d* = .46. Moreover, the difference between incongruent trials with a different response and incongruent trials with the same response was smaller in the small number of colors as compared to large numbers of colors, *t*(46) = 2.94, *p* = .002, *p*_holm_ = .005, *BF*_10_ = 6.82, *Cohen’s d* = .43.

Further analysis of the differences between the conditions across all the number of colors showed meaningful differences. While no differences were found between small and large numbers of colors for both congruent (*t*(46) = .7, *p* = .48, *pholm* = .96, *BF*_10_ = .2 ≡ *BF*_01_ = 4.98, *Cohen’s d* = .1) and incongruent trials with a different response (*t*(46) = 0.49, *p* = .63, *pholm* = .96, *BF*_10_ = .18 ≡ *BF*_01_ = 5.64, *Cohen’s d* = .07) conditions, for the incongruent trials with the same response condition, in the large number of colors condition, the RTs were faster than those in the small number of colors, *t*(46) = 2.57, *p* = .01, *pholm* = .04, *BF*_10_ =2.99, *Cohen’s d* = .37. Moreover, neutral trials with a large number of colors were much faster than those with a small number of colors, *t*(46) = 5.35, *p* < .001, *pholm* < .001, *BF*10 > 1,000, *Cohen’s d* = .78.

## Discussion

We conducted a color-digit Stroop task in which participants were presented with colored stimuli and were asked to decide whether the number of colors in the stimuli was smaller or larger than 5. The stimuli (an extension of Hershman, Keha, et al.’s (2024) set of stimuli) were either rectangles (the neutral condition) or digits that consisted of several horizontal slices of colors. The numerical value of the digits was either congruent or incongruent with the number of colors. Participants were asked to respond using two keys, one for smaller than 5 and one for larger than 5. Following De Houwer’s (2003) paradigm, the incongruent trials included both incongruent trials with the same response (e.g., the digit 9 consisting of eight colors) and incongruent trials with a different response (e.g., the digit 9 consisting of two colors). These two types of incongruent trials had been suggested to lead to two different incompatibilities. Specifically, 1) incongruent trials with the same response revealed SSC between the numerical information in the relevant and irrelevant dimensions, and 2) incongruent trials with a different response caused both SSC and SRC (Chen et al., 2011; De Houwer, 2003; Shichel & Tzelgov, 2018; van Veen & Carter, 2005).

The results of the present study revealed that when the number of colors was smaller than 5, incongruent trials with a different response were slower than incongruent trials with the same response, providing evidence of the existence of SRC. In addition, incongruent trials with the same response were slower than congruent trials. This is evidence of the existence of SSC. No meaningful difference was found between congruent and neutral trials.

Similar to a small number of colors, also when the number of colors was larger than 5, incongruent trials with a different response were slower than incongruent trials with the same response (evidence of the existence of SRC). However, in contrast, when the number of colors was larger than 5, no differences were found between incongruent trials with the same response and congruent trials. This means that no evidence of SSC was found. In addition, reverse facilitation was found so that congruent trials were slower than neutral trials, which is evidence of a task conflict (Littman et al., 2019).

A further analysis was conducted to find the source of the different effects found in stimuli containing smaller or larger than 5 colors. The analysis showed that congruent trials and incongruent trials with a different response showed similar RTs across a different number of colors (i.e., smaller or larger than 5). In contrast, both neutral and incongruent trials with the same response were statistically faster when the stimuli contained more than five colors.

The acceleration in responses for incongruent trials with the same response with large numbers of colors is evidence of different processing of the number of colors (and not due to interference from the congruent trials). When participants concluded that the number of colors was larger than 5, the exact number magnitude processing was stopped. That is, the exact number of colors was not detected, and therefore, no comparison between the numerical value of the stimulus and the exact number of colors that consisted of the number occurred. Therefore, a large number of colors (regardless of whether the exact number of colors will be similar or different to the numerical value of the stimulus) will be congruent with a large value of the digit that is composed of the colors. In other words, in a large number of colors, incongruent trials with the same response are similarly processed as congruent stimuli that have the same two pieces of information from different dimensions: the numerical value of the stimulus is larger than 5. This implies that in stimuli composed of a large number of colors, the exact number of colors that is not required for the decision is more easily ignored.

Interestingly, the same pattern is also observed with neutral trials (i.e., faster responses in large numbers compared to small numbers of colors). Similar to the incongruent trials with the same response, the exact number of colors is more easily ignored. However, in contrast to the incongruent trials with the same response, in the neutral trials, there is no numerical value to process. Therefore, we suggest that our findings provide evidence for two types of task conflict. The first task conflict refers to the determination of whether the number of colors is smaller or larger than five (the relevant task) and the processing of the numerical value (the irrelevant task). Second, and interestingly, our finding provides evidence for another novel task conflict that refers to the relevant task: determining whether the number of colors is smaller or larger than five (the actual relevant task) and the spontaneous processing of the exact number of colors (the secondary, irrelevant task). In other words, in addition to the frequently discussed task conflict that rests on the processing of competing stimulus dimensions (and thus, poses high demands on cognitive conflict processing), we revealed another type of task conflict that refers to the same aspect of the stimuli (the number of colors) but to an irrelevant task (i.e., deciding whether the number of colors is smaller or larger than 5 vs. processing the exact number of colors).

While evidence for the existence of information conflict (i.e., slower responses for incongruent trials than for congruent trials) is often found (Henik et al., 2018; MacLeod, 1991), as well as evidence for the existence of both SSC and SRC (Chen et al., 2011; De Houwer, 2003; Shichel & Tzelgov, 2018; van Veen & Carter, 2005), evidence for the existence of task conflict (i.e., faster responses for neutral trials than for congruent trials) might be observed by using manipulation of control (Goldfarb & Henik, 2007; Kalanthroff & Henik, 2013; Kinoshita et al., 2018) or by using sensitive measurements such as brain activation (Bench et al., 1993; Carter et al., 1995) or changes in pupil size (Hershman et al., 2021; Hershman & Henik, 2019). Alternatively, it was also suggested that it is possible to measure task conflict by models of behavioral data, either with ex-Gaussian analyses for RTs (Steinhauser & Hübner, 2009) or with multinomial processing tree models for error rates (Moretti et al., 2023). Here, in a Stroop-like task, we revealed a pattern that is different than the pattern in a similar word-color Stroop task. When participants were instructed to decide if the presented number of colors was smaller or larger than 5 and ignore the meaning of the object that was created by the presented colors, we found clear-cut evidence for the existence of information conflict and, moreover, of both SSC and SRC. In addition, evidence for the existence of task conflict was also found. It has already been suggested that task conflict should represent a major component of Stroop interference (Levin & Tzelgov, 2014). Therefore, our findings might be relevant to researchers who are interested in cognitive control in general and in task conflict in particular (Hershman et al., 2020; Littman et al., 2019).

Our findings suggest that certain aspects of the commonly used Stroop task are shared by all control situations regardless of the content area (e.g., words vs. numbers), whereas other aspects depend on the content area and the type of processing involved (e.g., small vs. large numbers). While the presence of a large number of colors might trigger estimation processes (rather than more effortful and, thus, slower, precise judgment procedures; Trick & Pylyshyn, 1994; for a review, see Mix et al., 2002), the presence of a small number of colors seems to elicit spontaneous but somewhat more time-consuming processing of number magnitudes. Furthermore, our findings suggest that the processing of a small number of colors may reflect a kind of subitizing process (Starkey & Cooper, 1980; Trick & Pylyshyn, 1994) or an object-tracking system targeted at discrete quantities (e.g., Feigenson et al., 2004; Xu, 2003), while the quicker estimation strategy applied to a large number of colors may reflect an estimation process (that is more likely to occur with continuous quantities).

In the standard two-to-one color Stroop task (De Houwer, 2003), there is no association between the stimuli that are assigned with the same response key (e.g., yellow and green to the “C” key, and red and blue to the “M” key). In contrast, in the present study, there is a clear association among the stimuli that are associated with the same response key (e.g., 1–4 are assigned to the ‘C’ key, while 6–9 are assigned to the ‘M’ key). This easy-to-follow association in the present study (i.e., all the mapped stimuli are smaller or larger than 5) does not require task-irrelevant cognitive resources for working memory (with the aim to remember an arbitrary mapping). Hence, it makes the present task appropriate for the examination of interference processing in populations that have difficulties with working memory (e.g., neurodevelopmental, neuropsychiatric, and neurological populations). In addition, since the task is language-independent (because reading is not required to complete the task), this task is appropriate for the examination of interference processing in pre-literate children and individuals (patients) with low/deficient reading proficiency.

## Summary

The present study makes use of the stimuli and the conceptual framework suggested by Hershman, Keha, et al. (2024). Participants were asked to respond to the number of colors and ignore the numerical value of the digit. Specifically, participants were asked to determine whether the number of colors is greater or smaller than 5 using two keys, one for smaller than 5 and one for larger than 5. This response setting enabled us to test two different processes, which were not possible to test in the original study, namely the SSC and SRC (see De Houwer, 2003). Moreover, the current study enabled us to dissociate between processes associated with judgments of precise numbers and those associated with the estimation of approximate numbers. Specifically, it allowed us to study two types of task conflict. One task conflict refers to the task-irrelevant processing of the numerical value of the stimulus (the task-irrelevant stimulus dimension) vs. determining whether the number of colors is smaller or larger than 5 (the task-relevant stimulus dimension). In addition, the task used in the present study allowed us to observe a task conflict that refers to the processing of the exact number of colors (the task-irrelevant properties) vs. the determination of whether the number of colors is smaller or larger than 5 (the task-relevant stimulus property).

The presented task is a two-to-one Stroop-like task with an easy-to-follow response mapping. Therefore, it is language-independent and appropriate for the examination of interference processing in populations that have difficulties with working memory, as well as pre-literate children and individuals with low/deficient reading proficiency.

## Data Accessibility Statement

We wish to thank Ms. Desiree Meloul for her helpful comments and useful input on this article. This study was not preregistered. Data for the experiment is publicly available on OSF at: https://osf.io/g2jau/?view_only=9aeca2f8736b414398d1fb11eda34e60.
